# The interprofessional collaboration between police and crisis response team in managing suicide-related cases in Singapore

**DOI:** 10.1192/j.eurpsy.2023.1064

**Published:** 2023-07-19

**Authors:** V. Chan, K. Ng, U. K. Shariffuddin, X. Tang

**Affiliations:** Institute of Mental Health, Singapore

## Abstract

**Introduction:**

The Crisis Response Team (CRT) is an interprofessional collaboration between the Singapore Police Force (SPF) and the Mental Health Helpline (MHH) of the Institute of Mental Health (IMH). Supported by a multidisciplinary team comprising of the SPF, IMH psychiatrists, community psychiatric nurses and crisis counsellors, and community partners, this intervention aims to support suicidal individuals, depending on their risk severity, residing in the community.

**Objectives:**

To present the CRT work process and to explore the characteristics and outcomes of suicide-related cases referred.

**Methods:**

In this descriptive research study, a quantitative approach is adopted. An Excel file shared across the helpline counsellors is used to collate information of the referred cases. Data collected from October 2021 to August 2022 were evaluated using the IBM SPSS Statistics for Windows v28.0. Descriptive statistics were used to summarise the characteristics and outcomes of the cases.

**Results:**

Figure 1 shows the CRT work process. To standardise the method of assessing both suicide ideation and behaviour, the Columbia-Suicide Severity Rating Scale (C-SSRS) is utilised. As compared to other suicide ideation and behaviour scales, the C-SSRS has demonstrated good convergent and divergent validity, high sensitivity and specificity for suicidal classifications, and moderate to strong internal consistency (Cronbach’s α: 0.73 - 0.95) (Posner et al. AJP 2011; 168(12) 1266-1277). A total of 3,386 suicide-related cases was referred. The age range of the suicide-related cases range from 8 – 97 years old (M = 36, SD = 17.33). Of these 3,386 cases, 627 cases were discharged back to their family members/employer/friend/partner and with follow-up check-in calls by the MHH counsellors, 416 cases were sent to the restructured hospitals for organic workup, 2,268 cases were brought back to IMH, 55 cases were discharged back to the SPF for further investigation, and 20 cases warranted CRT home visit activations. Figure 2 shows the total number of referred cases and outcome of these cases in each month.

**Image:**

**Figure:**
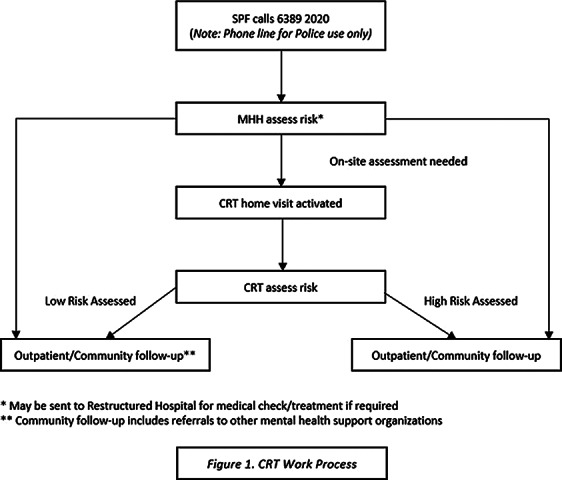

**Image 2:**

**Figure:**
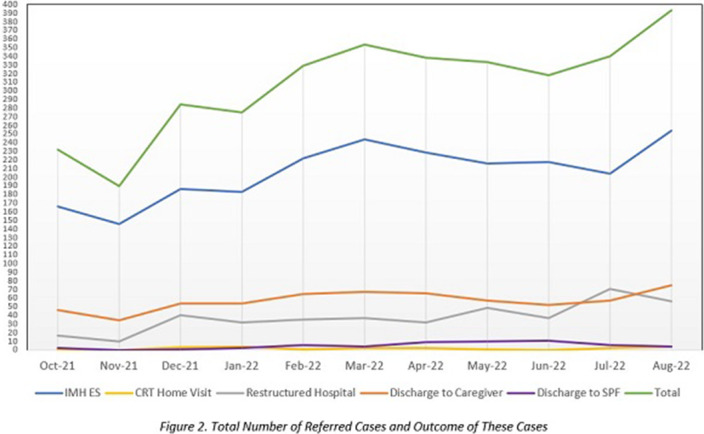

**Conclusions:**

The CRT intervention could mitigate suicide risk and pressure on the mental health system (i.e., reduce unnecessary emergency room visits and hospital admissions), create greater mental health awareness, and facilitate individuals’ connection to mental healthcare services (i.e., in hospitals and/or in the community) as evidenced by the increasing number of cases referred, and increased collaboration with the various stakeholders, ensuring timely intervention and necessary follow-ups thereafter.

**Disclosure of Interest:**

None Declared

